# Roles of *JnRAP2.6-like* from the Transition Zone of Black Walnut in Hormone Signaling 

**DOI:** 10.1371/journal.pone.0075857

**Published:** 2013-11-12

**Authors:** Zhonglian Huang, Peng Zhao, Jose Medina, Richard Meilan, Keith Woeste

**Affiliations:** 1 Department of Forestry and Natural Resources, Hardwood Tree Improvement and Regeneration Center (HTIRC), Purdue University, West Lafayette, Indiana, United States of America; 2 College of forestry, Northwest Agriculture and Forestry University, Yangling, Shaanxi, China; 3 Career of Socioeconomic Development and Environment, Zamorano University, Tegucigalpa, Honduras; 4 USDA Forest Service Hardwood Tree Improvement and Regeneration Center (HTIRC), Purdue University, West Lafayette, Indiana, United States of America; Cankiri Karatekin University, Turkey

## Abstract

An EST sequence, designated *JnRAP2-like*, was isolated from tissue at the heartwood/sapwood transition zone (TZ) in black walnut (*Juglans nigra* L). The deduced amino acid sequence of JnRAP2-like protein consists of a single AP2-containing domain with significant similarity to conserved AP2/ERF DNA-binding domains in other species. Based on multiple sequence alignment, *JnRAP2-like* appears to be an ortholog of *RAP2.6L* (At5g13330), which encodes an ethylene response element binding protein in *Arabidopsis thaliana*. Real-time PCR revealed that the *JnRAP2-like* was expressed most abundantly in TZ of trees harvested in fall when compared with other xylem tissues harvested in the fall or summer. Independent transgenic lines over-expressing *JnRAP2*-*like* in *Arabidopsis* developed dramatic ethylene-related phenotypes when treated with 50 µM methyl jasmonate (MeJA). Taken together, these results indicated that *JnRAP2-like* may participate in the integration of ethylene and jasmonate signals in the xylem and other tissues. Given the role of ethylene in heartwood formation, it is possible *JnRAP2-like* expression in the transition zone is part of the signal transduction pathway leading to heartwood formation in black walnut.

## Introduction

Heartwood formation is an important factor determining wood quality [1]. Black walnut (*Juglans nigra* L.) is one of the most valuable fine hardwood tree species in the Midwestern U.S. because of the excellent qualities of its heartwood. Despite its economic importance, heartwood formation is poorly understood at both the physiological and molecular level [2]. Most research on heartwood formation has focused on a narrow transition zone (TZ) located between the sapwood and heartwood [1,3,4] because the death of all parenchyma cells at the TZ of angiosperms results in the formation of heartwood [5-7]. Heartwood formation in black walnut probably occurs at the time of cambial dormancy in the fall [4,8-10], but it can also be understood as a developmental process involving a complex series of events that take place throughout the year [11]. Heartwood formation is strongly spatially regulated; in many ways it resembles programmed cell death (PCD) [5,12-14], but it is not clear how cell death in the TZ is related to the formation and deposition of extractives that produce the familiar colors of hardwood lumber [2,15,16], since not all tree species have darkly-colored heartwood. Extractives are primarily phenolic compounds polymerized to varying degrees; most colored heartwood is associated with the accumulation of polyphenols, flavonoids and isoprenoids [17]. In black walnut, the TZ can be identified macroscopically because it fluoresces blue under UV light [4], perhaps because of the accumulation of sinapic acid esters [18]. Endogenous and exogenous factors can affect heartwood formation, including phytohormones, photoperiod and temperature [17,19]. For example, ethylene is associated with heartwood formation [8,9], but the mechanism by which environmental and hormonal signals are interpreted at the TZ is not clear [2]. Cheong [20] proposed a general pathway connecting wounding, secondary metabolism, and senescence. Kato [21] showed a connection in *Cucurbita maxima* between wounding, ethylene, ACC oxidase and the induction of phenylalanine ammonia-lyase, the first enzyme in the phenylpropanoid pathway. Hudgins and Franceschi [22] demonstrated that methyl jasmonate was far more effective in the induction of phenolic defenses in conifer xylem than wounding. They also showed that the differentiation of resin-secreting cells in conifer xylem after treatment with methyl jasmonate was mediated by ethylene, which was perceived in xylem parenchyma cells, the primary cellular location of ACC Oxidase in mature xylem. ACC oxidase is the enzyme that catalyzes the final step in ethylene biosynthesis.

The APETALA2/ETHYLENE RESPONSE FACTOR (AP2/ERF) super-family, one of the large multiple-gene families of transcription factors unique to the plant kingdom, is characterized by the presence of the AP2/ERF DNA-binding domain, which consists of about 60 to 70 conserved amino acids. This super-family has been subdivided into three subfamilies (AP2, ERF, and RAV) based on their sequence similarities and numbers of AP2/ERF domains [23]. The ERF subfamily has only one AP2/ERF domain [23,24] that mediates plant responses to biotic and abiotic stress through binding to a *cis*-acting element, the GCC box [25,26]. The GCC box is especially associated with genes involved in ethylene responses [27]. There are a large number of ERF subfamily members (> 100) in all well-characterized genomes [23,28]. *RAP2.6-like* (RAP2.6L, At5g13330, NCBI RefSeq NP_196837, *ERF113*) is a member of the B4 subgroup of the ERF/AP2 transcription factor family. In *Arabidopsis*, *RAP2.6L* is expressed most strongly in mature, dried seeds, mature flowers (especially stamens), hypocotyls, the maturation zone of roots and in developing xylem [29,30] (http://www.bar.utoronto.ca/efp/cgi-bin/efpWeb.cgi). A poplar ortholog of *RAP2.6L*, PtpAffx. 75787.2.A1_s_at (= fgenesh4_pg.C_LG_1000582) was very highly expressed in male and female flowers, and highly expressed in xylem and roots [31]. A second poplar ortholog (eugene3.00031319) was highly up-regulated in response to the onset of short days and appears to regulate a transcriptional cascade at the onset of dormancy [32]. In *Citrus*, a putative homolog of *RAP2.6L* is upregulated during ethylene-promoted laminar abscission [33]. *RAP2.6L* also regulates expression programs during shoot, root and callus development of *Arabidopsis* during tissue culture. When explants were placed on shoot-inducing media (containing elevated cytokinin/auxin ratios), *RAP2.6L* played an early and pivotal role in regulating subsequent gene expression [34]. Its expression preceded organ primordial formation. Indeed, its expression may be induced quickly and strongly by cytokinin [35], although others found only a slight induction by trans-zeatin and a more pronounced induction by ABA, and a strong induction by NaCl [29,36]. *RAP2.6L* is also upregulated by biotic stress, specifically by infiltration of *Pseudomonas syringae* [29], and by abiotic stress, specifically in response to 40 µM selenate [37]. 

Genes in the ERF subfamily from black walnut have not yet been identified. Here we first report on an AP2 domain-containing transcription factor, *JnRAP2-like*, expressed in the TZ of black walnut. Expression of this gene in black walnut was investigated with regard to stage of development, seasonal changes, and tissue type, and its putative functions were reinforced by over expression (OE) studies in *Arabidopsis*. 

## Materials and Methods

### Plant Materials and Growth Conditions

#### Black Walnut

**Figure 1:**
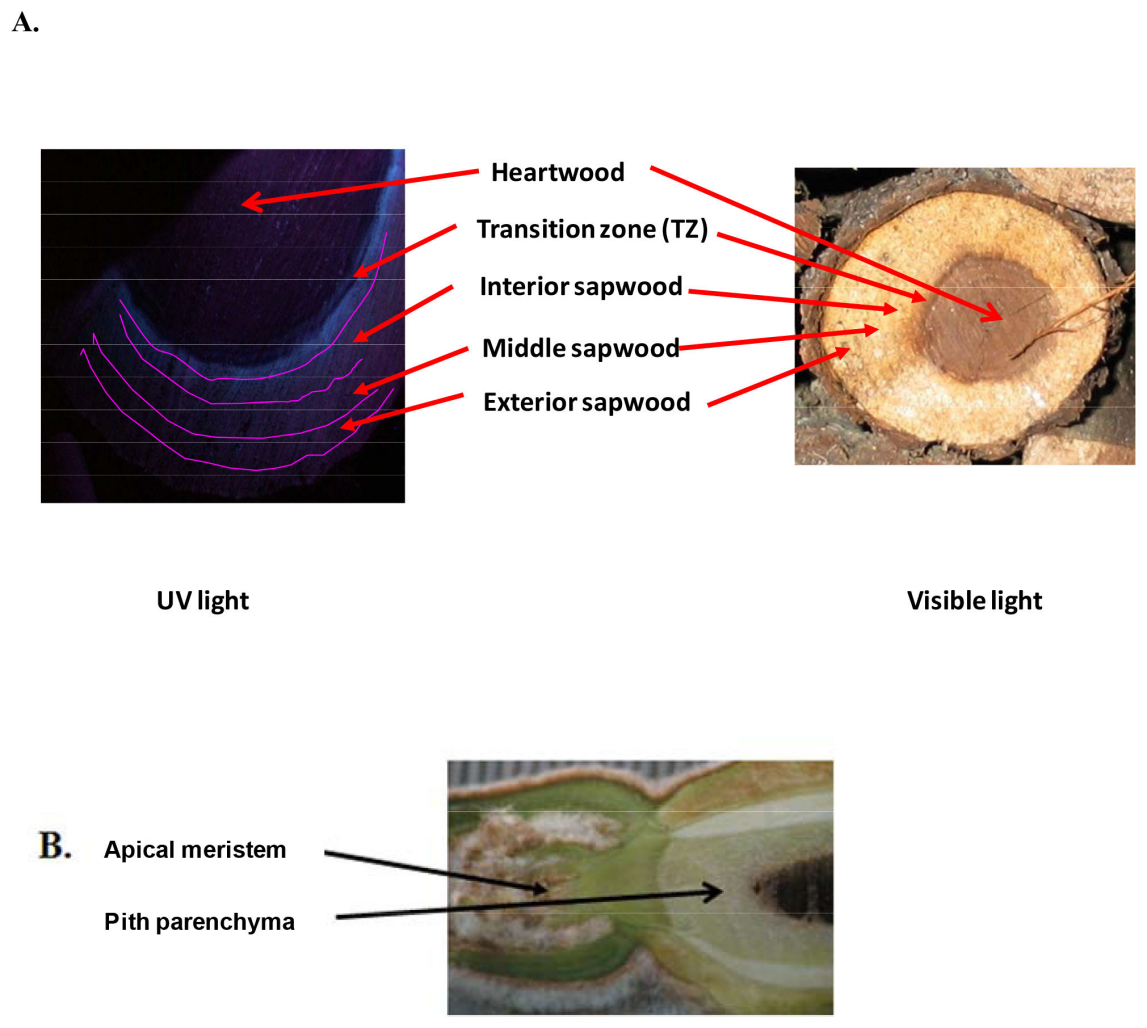
The pictures of a black walnut stem and the pith parenchyma. **A**. Cross-section of a black walnut stem under UV and white light. The TZ fluoresces blue under UV light; the interior sapwood is defined by ~ 1 cm away from the TZ; the middle sapwood and exterior sapwood are divided evenly from cambium region (light green color under visible light) to the interior sapwood. **B**. The location of the pith parenchyma in a branch of black walnut. Scale bar = 1 mm. Note: The trees in this study belong to HTIRC at Department of Forestry and Natural Resources. No specific permits were required for the described field studies. No specific permissions were required for the locations/activities. The location in this study is not privately-owned or protected in any way. The field studies in this research did not involve endangered or protected species.

Two thirty-nine-year-old black walnut trees grown at the Martell Research Forest, Tippecanoe County, IN, were cut down on July 1 and October 14, 2004. These trees were labeled “summer tree” and “fall tree”, respectively. Another four black walnut trees were felled on the same dates in 2006. Immediately after the trees were felled, stem cross-sections (“cookies”), approximately 2.5 cm thick and 20 cm in diameter, were cut with a chainsaw. The cookies were immediately submerged in liquid nitrogen. After returning to the lab, the cookies were transferred to an ultra-low freezer (-80 °C) for storage. TZs were identified under UV light [4] and carefully chiseled out of the cookies from each tree. Multiple cookies from a single tree were used to produce RNA; multiple extractions were required to obtain sufficient RNA for use as a single replicate. Other tissues including interior sapwood, exterior sapwood, and vascular cambium /phloem (all green tissues inside of the bark and outside of the xylem) from these six trees harvested from summer and fall were also removed from the frozen cookies harvested as described above (Figure 1A) [38]. Roots were collected from about ten young walnut trees growing in a greenhouse, RNA was extracted from each separately. Embryogenic calli were from in vitro cultures of a single source genotype; RNA was extracted from multiple plates of calli and pooled. Pith parenchyma tissues were collected from multiple branches of three genotypes and the RNA extracted from the pooled tissue sample. RNA from female flowers, male flowers, green leaves, and partially and fully senescent leaves were obtained by pooling multiple samples taken from many branches of a 15-year-old black walnut tree growing on the Purdue University campus. Senescent leaves were sampled in late summer and early autumn. The region of the pith parenchyma was identified as the tissue subtending the apical meristem superior to the pith (Figure 1B) and inside the vascular cylinder. RNA was isolated as described previously [39]. Xylem tissue was ground to a fine powder in a 6750 SPEX CertiPrep freezer mill (SPEX CertiPrep, INC; Metuchen, NJ).

### Identification and the selection of the *JnRAP2-like*

Previous research showed that heartwood formation may be related to signaling pathways, such as ethylene, abscicic acid, and jasmonate dependent signaling pathways. A cDNA library has been made by Dr. Christian Breton (INRA Unité Amélioration, F4516 Olivet Cedex, France) from TZ tissue of black walnut. Based on these two factors, 78 partial gene fragments including the putative transcription factors have been selected and semi-quantitative RT-PCR was further performed to screen the differentially expressed genes. Another reference can be found in http://link.springer.com/article/10.1007/s11105-009-0144-x/fulltext.html.

A partial sequence of *JnRAP2-like* from this cDNA library was used to obtain the full-length coding sequence of *JnRAP2-like* by 5’and 3’RACE. *JnRAP2-like* cDNA is 678 bp (Accession No.: FJ830598) and encodes a putative protein of 226 amino acids with a predicted molecular weight of 25.12 kD. 

### *Arabidopsis* (Columbia-0) Plants

Plants were grown under a 16-hr photoperiod per day at a constant temperature of 25 °C either on Murashige and Skoog (MS) [40] basic medium with 2% sucrose and 0.22% phytogel, or in soil. 

### RNA isolation

RNA from black walnut trees was isolated as described previously [39]. Xylem tissue was ground to a fine powder in a 6750 SPEX CertiPrep freezer mill (SPEX CertiPrep, INC; Metuchen, NJ). Extraction buffer consisted of the following: 200 mM Tris, pH 8.5, 1.5% lithium dodecyl sulfate, 300 mM lithium chloride, 10 mM disodium salt EDTA, 1% sodium deoxycholate, 1% tergito powder P-40, 5 mM thiourea, 1 mM aurintricarboxylic acid, 10 mM dithiothreitol, 2% polyvinylpolypyrrolidone, and 2% ß-mecaptoethanol. RNA extraction from *Arabidopsis* seedlings was carried out using Qiagen™ plant RNA isolation kits. RNA concentration was measured with a NanoDrop 1000 spectrophotometer (Wilmington, DE). 

### Isolation of Full-length cDNA by 5′- and 3′-RACE

The SMART RACE cDNA amplification kit (Clontech; Mountain View, CA) was used to perform 5’- and 3’-RACE following the manufacturer’s instructions. Samples of total RNA were used for reverse transcription. The gene-specific primer GSP1 (Table 1) designed from the antisense strand, was used for 5′-RACE, and GSP2 (Table 1) from the sense strand was used for 3′-RACE. All RACE reactions were performed using the following PCR protocol: 94 °C for 30 s, 68 °C for 30 s and 72 °C for 3 min for 30 cycles. The PCR product was subcloned into pGEM-T vector (Promega; Madison, WI) and recombinant clones sequenced. The contigs were aligned with Sequencer TM 4.1 (Gene Codes; Ann Arbor, MI). 

**Table 1:** Primers used in this study.

Primer Name	Sequence (5' to 3')
18s rRNA Forward	5' AGAGGCCTACAATGGTGGTG 3'
18srRNAReverse	5' CCTCCAATGGATCCTCGTTA 3'
GSP1 for 5' RACE	5' AGTCTGGCTCGGCACATTCGAAACC
GSP2 for 3' RACE	5' GGTTTCGAATGTGCCGAGCCAGACT
RAP2-like_Forward_for_real-time PCR	5' CGAAATTCGAGACCCAAAAA 3'
RAP2-like_Reverse_for_real-time PCR	5' CTGGGAGACGCTCGTATTTC 3'
RAP2-like_35S_Forward_for_pBI121	5' ATCGGGTACCTGTGGAATTGTGAGCGGA 3'
RAP2_like_35S_Reverse_for_pBI121	5' ATCGGGTACCAAACGACGGCCAGTGAAT 3'
KAN_Forward_for QD-PCR	5' CTGTGCTCGACGTTGTCACT 3
KAN_Reverse_for QD-PCR	5' AGCCAACGCTATGTCCTGAT 3'
4HPPD_Forward_for QD-PCR	5' GCGCTTCCATCACATCGAGTTC 3'
4HPPD_Reverse_for QD-PCR	5' AATCCAATGGGAACGACGACGC 3'
PetC_Forward_for QD-PCR	5' TAAGACTCATGGTCCCGGTGAC 3'
PetC_Reverse_for QD-PCR	5' ACCATGGAGCATCACCAGTCCT 3'
At5g13330-Forward	5' TCGGATCAACATCAACCAGA 3'
At5g13330-Reverse	5' GTTTAGCCTTGGTGCCTTTG 3'
RAP2-like_Full_ORF-Reverse	5' TGGGATTGCTAGAATTGACGCCC 3'
RAP2-like_Full_ORF-Forward	5' TACTGACGACATGTCTGCCATGG 3'
PDF1.2 _Forward	5' AATGAGCTCTCATGGCTAAGTTTGCTTCC 3'
PDF1.2 _Reverse	5' AATCCATGGAATACACACGATTTAGCACC 3'
ChiB _Forward	5' GCTTCAGACTACTGTGAACC 3'
ChiB _Reverse	5' TCCACCGTTAATGATGTTCG 3'
TUB _Forward	5 CGGAATTCATGAGAGAGATCCTTCATATC 3'
TUB _Reverse	5' CCCTCGAGTTAAGTCTCGTACTCCTCTTC 3'
GAPC-Forward	5' TCCCGTGTGGTCGACTTGA 3'
GAPC-Reverse	5' CCACTCCCTATCATTCGAGATCTG

### Multiple Alignment and Phylogenetic Tree Construction

Putative orthologs of *JnRAP2-like* in different plant species, including *Capsicum annuun*, *Lotus japonicas*, *Brassica oleracea*, *Mesembryanthemum crystallinum*, *Malus* × *domestica*, *Medicago truncatula*, *A. thaliana*, *Oryza sativa*, and *Nicotiana tabacum*, were selected based on the results from Basic Local Alignment Search Tool (BLAST) and their sequences were multiply aligned by using Clustal W [41] with the purpose of discerning the AP2 domain. A phylogenetic tree of closely-related proteins was constructed using COBALT [42] running on the National Center for Biotechnology Information website (http://www.ncbi.nlm.nih.gov/tools/cobalt/cobalt.cgi?link_loc=BlastHomeAd). 

### Construction of Plasmids and *Arabidopsis* Transformation

The coding sequence of *JnRAP2-like* (obtained by RACE) was cloned into a modified pBluescript T vector provided by Dr. Clint Chapple at Purdue University, digested with *Xho* I and *Sac* I, and then ligated into pBI121 vector, also provided by Dr. Clint Chapple, to generate the pBI121+*JnRAP2* construct. The recombinant plasmid was introduced into *Agrobacterium tumefaciens* strain GV 3850, and then into *Arabidopsis* Col-0 plants using the floral-dip method [43] in order to generate *JnRAP2-like* OE lines. Transformed plants were selected on the basis of their resistance to kanamycin. The integration of the transgene in selected lines was confirmed by polymerase chain reaction (PCR), quantitative dual target PCR (QD-PCR) [44], segregation analysis, and reverse transcription-PCR. 

Seeds were first treated with 70% ethanol for 60 sec, and then with 20% (v/v) commercial bleach containing 0.05% Tween-20 for 5 min, followed by three rinses with sterile deionized water. Afterwards, seeds were held at 4 °C for 2-4 days for vernalization, and transferred to MS basic medium containing the appropriate hormones (Table 2). After the indicated number of days, the phenotype of the seedlings was observed and photographed. Hypocotyl and root lengths were measured using ImageJ software (http://rsb.info.nih.gov/ij/). Seedlings from these experiments were harvested and frozen in liquid nitrogen for DNA or RNA extraction. DNA or RNA was extracted from two to three biological replicates of 15 to 20 plants each using RNeasy Mini Kit (Qiagen; Valencia, CA, USA).

**Table 2:** Hormones and treatment conditions used to evaluate phenotypes of *JnRAP2-like* over-expressing transformed lines.

Hormones	Form	Concentration	Days of Treatment	Plant Grown in Light or Dark
ACC	1-Aminocyclopropane-1-carboxylic acid	0 μM	5	Dark
		25 μM		
		50 μM		
Methyl Jasmonate	Methyl-jasmonate	0 μM	21	Light
		10 μM		
		25 μM		
		50 μM		
		200 μM		
ABA	Abscisic acid	0.1 μM	5	Light
Auxin	Indole-3-butyric acid	10 μM	7	Light
ACC+Jasmonate	1-Aminocyclopropane-1-carboxylic acid+Methyl-jasmonate	10 μM ACC+25 μM MeJA	21	Light

### Semi-quantitative RT-PCR

To analyze transcript abundance, total RNA was incubated with DNase (Ambion, Foster City, CA) at 37 °C for 30 min, followed by the addition of 5 μl DNase Inactivation solution (Ambion, Foster City, CA) at room temperature. After 2 min, the mixture was spun for 2 min full-speed in a table-top centrifuge at room temperature, and the supernatant was recovered for reverse transcription (RT). 18S rRNA was used as the internal standard for normalization. RT-PCR primers were designed with the aid of the web-based program “Primer3” (http://www.frodo.wi.mit.edu/cgi-bin/primer3/primer3_www.cgi), and potential inhibitory secondary structures of primers and the predicted amplicon were checked using the web-based program “mFold” [45] (http://www.bioweb.pasteur.fr/seqanal/interfaces/mfold-simple.html). Primers (Table 1) were designed to produce amplicons around 150 to 200 bp in size. First-strand cDNA was synthesized as described by the manufacturer (Invitrogen; Carlsbad, CA). cDNA derived from total RNA was added to PCR reactions consisting of 1X reaction buffer, 100 μM MgCl_2_, 100 μM dNTP’s, 10 pmol each primer, 0.25 U of TaqGo polymerase (Promega; Madison, WI) in a total volume of 25 μl. The thermocycler program consisted of a 2-min denaturation step at 94 °C, followed by 24 cycles of 94 °C (30 s), annealing at appropriate time (30 s), 68 °C (45 s), followed by a 10-min extension at 68 °C. Amplified products were electrophoresed through 1% agarose gels, detected by ethidium bromide staining, and photographed under UV light (UVP, BioDoc-It; Upland, CA). 

### Real-time PCR

We used an iQ^TM^ SYBR Green Supermix and the iQ5 Multicolor real-time PCR Detection System (Bio-Rad; Hercules, CA). Control (18S rRNA) and samples from black walnut and *Arabidopsis* were run in triplicate and repeated twice (technical replicates). The biological replicates (3) were from different “cookies” harvested in summer and fall of 2004 and 2006. Each 25-μl reaction consisted of 12.5 μl of SYBR Green PCR Master Mix (Bio-Rad), 10 pmol of each primer, and cDNAs derived from total RNA. The reaction protocol was 95 °C for 10 min, followed by 40 cycles at 95 °C for 15 s, 53~58 °C for 30 s, and 72 °C for 30 s, and an extension phase of 81 cycles of melt-curve analysis as described by the manufacturer (BioRad). Fold change of gene expression relative to the standard (18S rRNA) was defined by the formula 2^-ΔΔCt^ (comparative C_T_ method) (User's Manual, ABI PRISM 7700 Sequence Detection System, Perkin-Elmer Applied Biosystems), where ΔC_T_ = C_T_ (sample) − C_T_ (18S rRNA), ΔΔC_T_ = ΔC_T_ of samples -ΔC_T_ of the TZ harvested from fall. 18S rRNA has been shown to be a robust standard for reverse transcription qPCR [46]. C_T_-values are the number of PCR cycles at which signal significantly rises above background; a consistent C_T_ was applied across all replicates. All samples appearing in a single figure were analyzed simultaneously in a single qPCR run, and then RNA from a separate pool was analyzed as a second replicate, and so on. 

### DNA Isolation

Fresh plant tissues (1 g) was ground in 10 ml of DNA extraction buffer [50 mM TrisCl, pH 8.0, 20 mM EDTA, 1.4 M sodium chloride, 0.4 M lithium chloride, 2% cetyltrimethylammonium bromide (CTAB), 2% polyvinylpyrrolidone (PVP), 2% SDS] using a mortar and pestle. Two percent (v/v) ß-mercaptoethanol and 0.1 g PVP were added to each sample before incubation at 65 °C for at least 1 hr with periodic shaking. Two chloroform extractions were performed, followed by one extraction with phenol/chloroform/isoamyl alcohol (25:24:1), pH 8.0, and two additional chloroform extractions. The supernatant was transferred to a clean tube and 0.9 volume of cold isopropanol and 0.1 volume 3 M sodium acetate were added. After gentle swirling, the DNA was precipitated by centrifugation for 15 min full-speed in a table-top centrifuge at 4 °C. The pellet was washed with 1 ml of 70% ethanol followed by 10 min centrifugation at full-speed in a table-top centrifuge at 4 °C. Pellets were air dried and then dissolved in 100 μl TE buffer, pH 8.0, or sterile deionized water. RNase A (10 µg/ml) was added to each sample followed by incubation at 37 °C for 30 min. DNA extraction from *Arabidopsis* seedlings was carried out using Qiagen™ DNeasy Plant Mini Kits. DNA purity was checked by absorbance ratios (260/280 nm). DNA quality was evaluated electrophoretically.

### Southern Blot

Southern hybridization was used to determine the copy number of *JnRAP2-like* in the genome of black walnut. The probe was a fragment of *JnRAP2-like* cDNA containing the putative AP2 homeodomain (about 700 bp in length). The probe was labeled using AlkPhos Direct kit (GE Healthcare; Piscataway, NJ). Twenty-five micrograms of genomic DNA were digested overnight with *Eco* RV and *Xho* I and run in a 1% agarose gel in 1X TAE, before alkaline transfer onto a nylon membrane (Hybond N+, Ambion; Foster City, CA). Hybridization occurred overnight at 55 °C, followed by high stringency post-hybridization washes at 55 °C. Visualization of the hybridization product was performed by incubating the membrane with a chemiluminescent substrate (CDP Star, GE Healthcare; Piscataway, NJ), followed by overnight exposure to X-ray film (Biomax, Kodak; Sigma; St. Louis, MO). (Figure S1)

## Results

### Isolation and Characterization of *JnRAP2-like*

**Figure 2:**
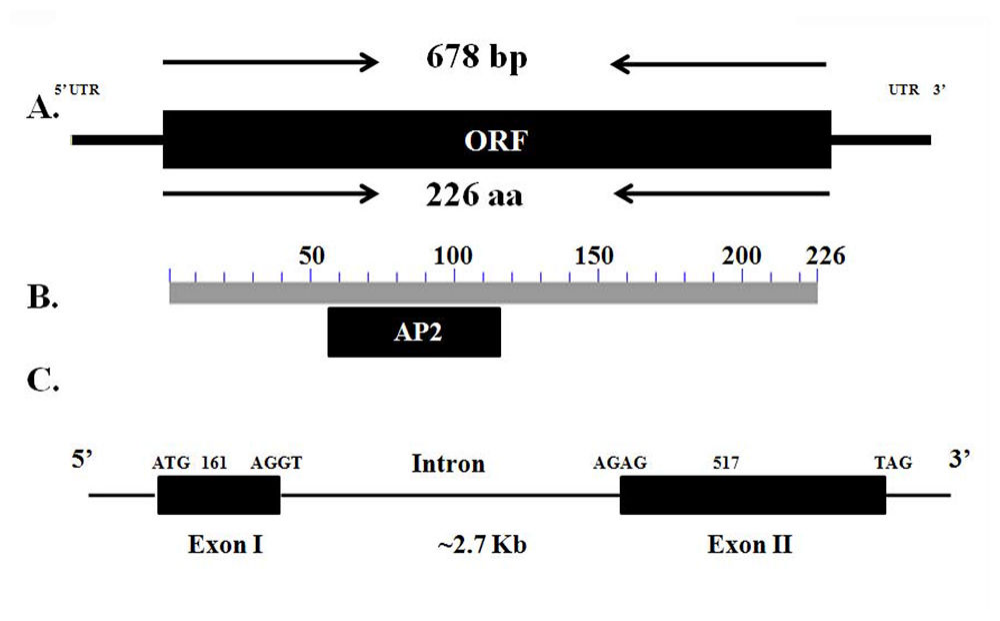
Structure of the *JnRAP2-like*. **A**. The full-length coding sequence of *JnRAP2-like* is 678 bp, starting with ATG and ending with TAG. **B**. The location of the AP2 domain. **C**. Two *JnRAP2-like* exons flank one poly-T-rich intron with splicing donor site (GT) and acceptor site (AG).

**Figure 3:**
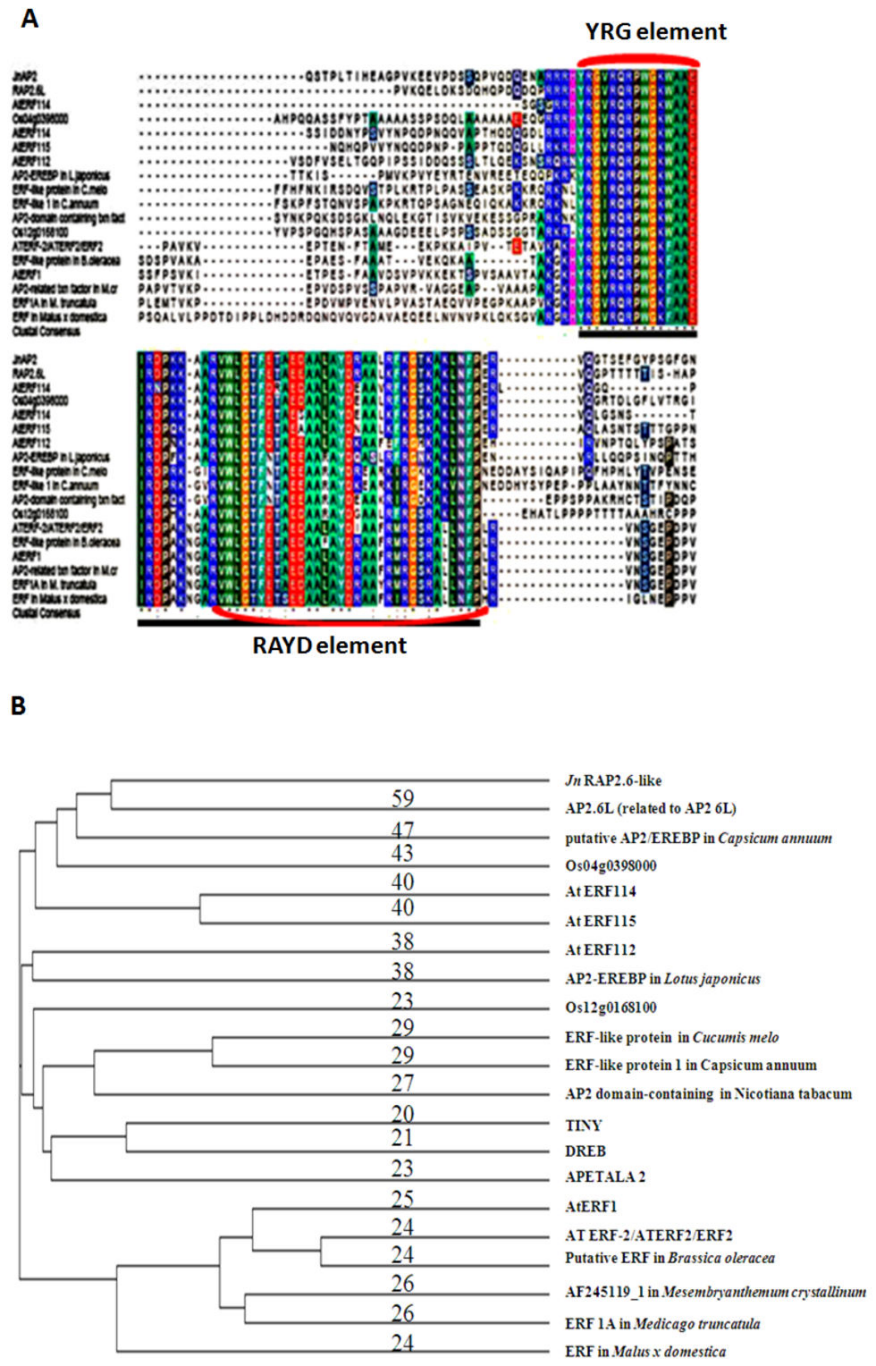
Deduced amino acid sequence for JnRAP2-like. **A**. Alignment of the AP2 domain of JnRAP2-like with proteins of other species. The consensus sequence derived from the alignment is underlined and invariant residues are indicated as * in the consensus. The bold, underscored amino acids represent an AP2 domain from amino acids 58 (Y) to 114 (P) including the YRG and RAYD elements. **B**. A phylogenetic tree relating JnRAP2-like protein to similar proteins produced using the software COBALT[42] (Papadopoulos and Agarwala, 2007).

To investigate the structure of *JnRAP2-like*, we isolated the genomic sequence of *JnRAP2-like* using a forward primer spanning the translational start site and a reverse primer spanning the translation stop site (Figure 2). The size of the genomic sequence of *JnRAP2-like* was about 2.7 Kb, with two exons flanked by the typical splicing donor (GT) and acceptor sites (AG), and a poly-T-rich intron (Figure 2). Analysis of the deduced amino acid sequence revealed that this protein has a single AP2 DNA-binding domain, indicating that *JnRAP2-like* may be a member of the ERF family of the AP2/ERF super-family (Figure 3A). *JnRAP2-like* shared a higher similarity with ERF proteins than with DREB (ABA39426.1) (23%), TINY (20%), or APETALA2 (21%) proteins, further evidence that *JnRAP2-like* is very likely an ERF-family gene. JnRAP2-like putative protein contains an AP2 DNA-binding domain from amino acid Y58 to P114 (Figure 3B), an alanine-rich region of 90 amino acids (A70-A109), but not a C-terminal serine-rich region found in many other ERF genes. The AP2 domain of *JnRAP2-like* also includes an YRG element that consists of 19-22 amino acids, and a RAYD element that consists of about 44 amino acids. The two domains are predicted to form an amphipathic α-helix. The YRG and RAYD elements may play a critical role in the structure and function of AP2 domain-containing proteins [47]. A putative C-terminal nuclear-targeting motif “QRVLN” was not found in JnRAP2-like [47]. Over the full amino acid sequence, JnRAP2-like shared 59% similarity with RAP2.6L (Related to AP2.6L, At5g13330), which is a member of the B-4 subgroup in the ERF family, but almost 100% identity within the AP2 domain (Figure 3B), evidence that *JnRAP2-like* is an ortholog of *Arabidopsis RAP2.6L*. 

### *JnRAP2-Like* was Highly Expressed in the TZ of Black Walnut

**Figure 4:**
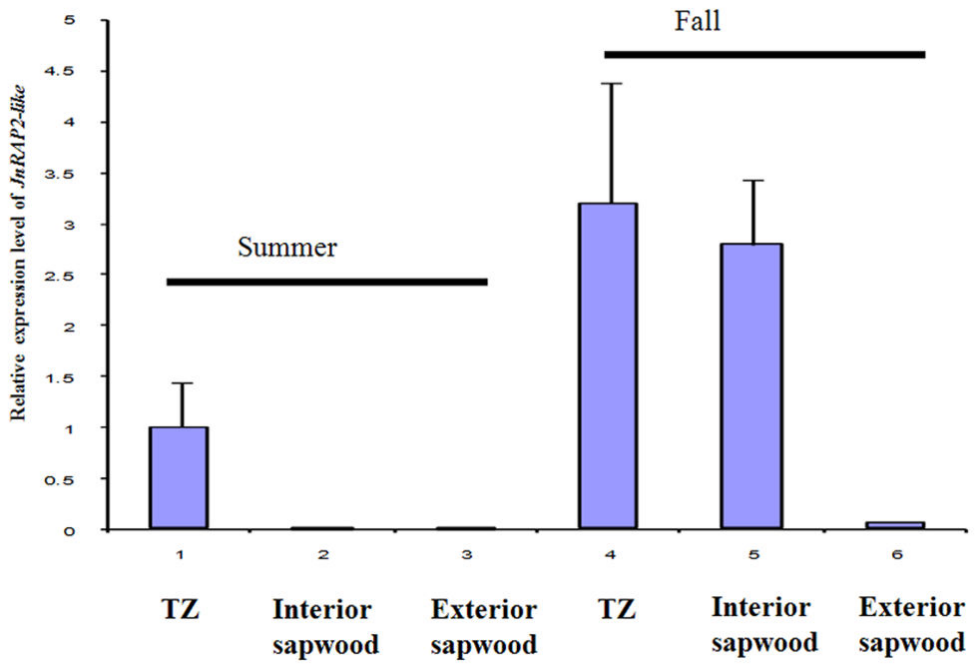
Transcript level of *JnRAP2-like* in the TZ, interior sapwood, and exterior sapwood from summer and fall trees. The fold changes were quantified by real-time PCR and were analyzed by the comparative C_T_ method by comparison with the *JnRAP2-like* transcript level in the TZ of the summer tree. Values are the means ± SE for three biological replicates. 18S rRNA was used as a standard.

The sapwood portion of the black walnut logs harvested in summer and fall was divided into four zones in order to investigate the expression of *JnRAP2-like* in more detail (Figure 1). Real-time PCR was performed, and results revealed that in the tissues harvested in summer, *JnRAP2-like* was mostly expressed in TZ and very weakly expressed in interior and exterior sapwood. *JnRAP2-like* expression was about 3 fold higher in the TZ in the fall when compared to the TZ harvested in summer (Figure 4), and was more than 3 times more abundant in the TZ in October versus other tissues, including interior sapwood harvested in summer, and exterior sapwood of trees harvested in both summer and fall (Figure 4). Expression of *JnRAP2-like* was similar in the TZ and inner sapwood of the tree harvested in fall. Because heartwood formation is thought to occur in autumn [4,9,10,15], the observed increase in expression of *JnRAP2-like* in xylem tissues harvested in fall relative to other tissues and other seasons may indicate it is associated with heartwood formation, possibly by influencing the transcription of genes in response to environmental signals such as shorter days or cold temperatures. 

### Family Members of *JnRAP2-like* in Black Walnut

To examine the copy number of *JnRAP2-like* in black walnut, two restriction enzymes (*Xho* I and *Eco* RV) were used to digest genomic DNA. The entire coding region of *JnRAP2-like* was used as a probe. We observed only one band in the DNA blots (Data not shown) and tentatively concluded that *JnRAP2-like* is a single-copy gene in the black walnut genome, as is *RAP2.6L* in *Arabidopsis*. 

### Expression of *JnRAP2-like* in Black Walnut Tissues

**Figure 5:**
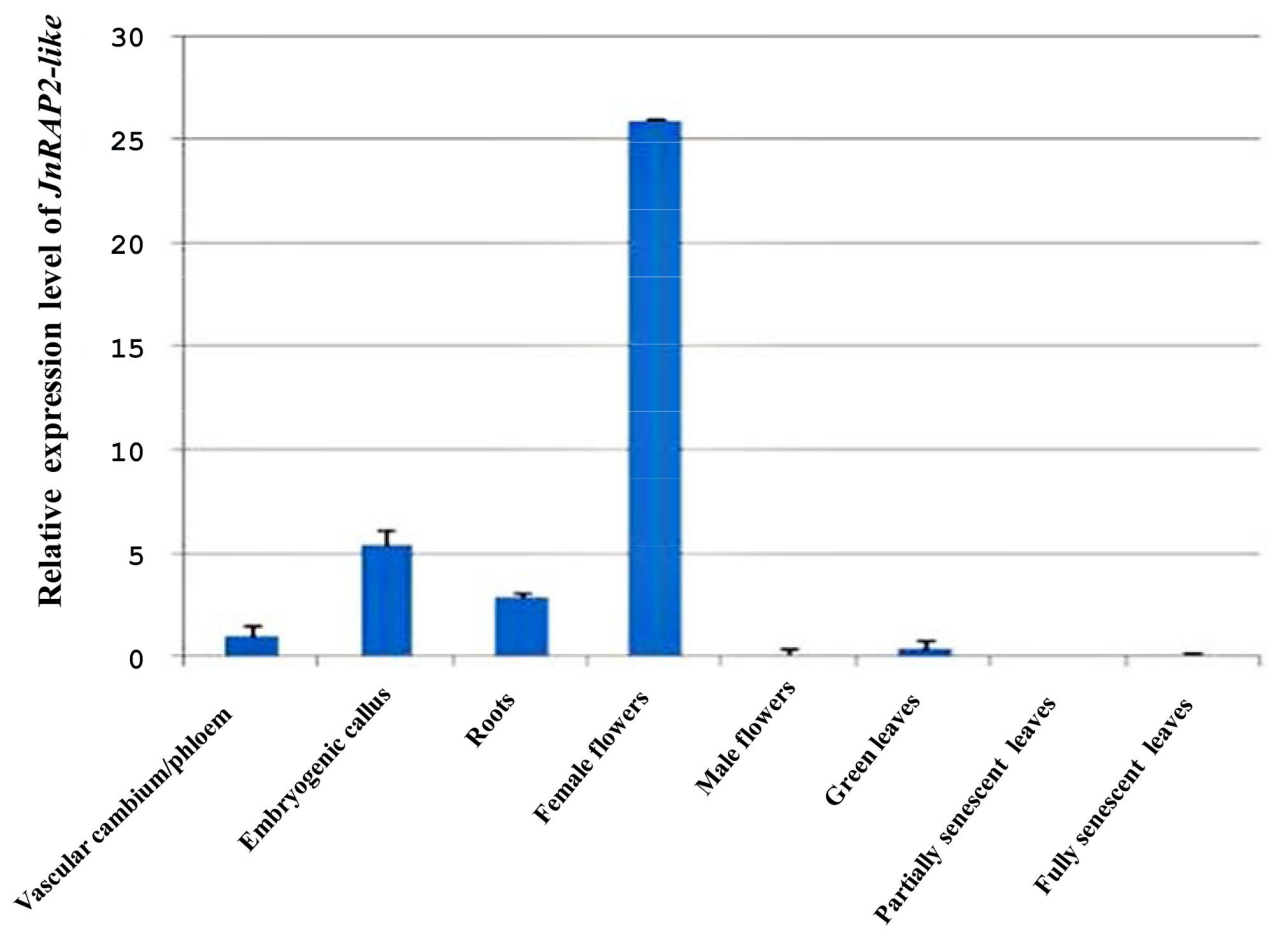
Transcript abundance of *JnRAP2-like* transcription factor in tissues of black walnut. Quantification of *JnRAP2-like* expression in black walnut tissues using real-time PCR. 18S rRNA was used as a standard. The fold changes were quantified and analyzed by the comparative C_T_ method. Values are the means ± SE for two biological replicates.

To understand the role of the *JnRAP2-like* in tree growth and development, we examined its expression in a series of tissues, including the region containing the pith parenchyma, vascular cambium/phloem (harvested in fall, 2004), embryogenic calli, roots, female and male flowers, green leaves, and partially and fully senescent leaves. Total RNA was extracted from these tissues. Conventional RT-PCR was first performed to test the expression of *JnRAP2-like* in each tissue, and results showed that *JnRAP2-like* was detectable in vascular cambium/phloem, embryogenic calli, roots, and female flowers, but not detectable in the tissues containing pith parenchyma, male flowers, green leaves, and partially and fully senescent leaves (data not shown). Real-time PCR was used to quantify the transcript amount in these tissues. Results showed that *JnRAP2-like* was expressed 26-fold higher in female flowers, 5.5-fold greater in embryogenic calli, 2.9-fold greater in roots, but only 0.38 times as much in green leaves, when compared to the expression in vascular cambium/phloem (Figure 5). These results indicate that *JnRAP2-like* very likely has functions outside xylem development, including female flower development, as was observed for the *Arabidopsis* and poplar orthologs [29]. The high abundance of *JnRAP2-like* in female but not male flowers was surprising because it contrasts with the observed expression of the putative poplar ortholog PtpAffx.5787.2.A1_s_at [29], which was strongly expressed in both male and female flowers. The same poplar ortholog was also strongly expressed in xylem and roots.

### Phenotypes of Transgenic Plants Over-expressing *JnRAP2-like* in *Arabidopsis*

**Figure 6:**
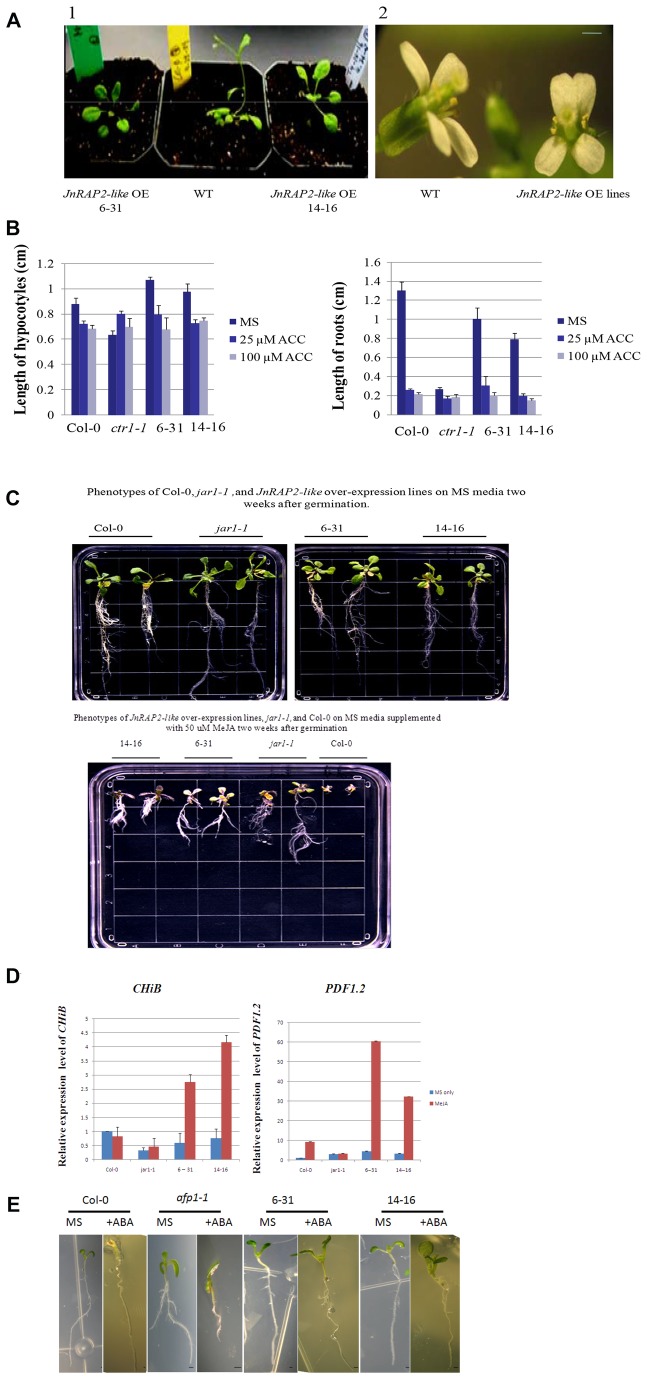
Responses of *JnRAP2-like* over-expression (OE) lines to hormone treatment. **A**. (1) Phenotypes of wild-type and *JnRAP2-like* OE plants. (2) Morphology of wild-type plant flowers (left) and *JnRAP2-like* OE plant flowers (right). **B**. Lengths of hypocotyls and roots of wild-type and *JnRAP2-like* OE plants. *ctr1-1* was used as a ethylene-response reference. **C**. Phenotype of two representative lines overexpressing *JnRAP2-like* in the wild type (Col-0) background compared to Col-0 and *jar1-1* (JA-response mutant) plants. Top, all plants grown for two weeks on MS only; bottom, all plants grown for three weeks on MS containing 50 µM MeJA. **D**. Expression of defense-associated genes *PDF1.2* and *ChiB* in *JnRAP2-like* OE lines and wild-type plants with and without 50 µM MeJA treatment two weeks after germination. Gene expression is presented relative to average treated wild-type levels. The average and SE of two biological replicates are presented. **E**. Phenotype of two representative lines overexpressing *JnRAP2-like* compared to Col-0 and *afp1-1*, ABA-response marker plants. All plants were grown for one week on MS containing 0.1 µM ABA. Scale bar = 1 mm.

After analyzing the expression of *JnRAP2-like* in 30 independent transgenic lines, we selected for further functional analysis two independent, single-copy, homozygous T3 lines (6–31). These OE lines displayed phenotypes similar to wild-type (WT) plants on half-strength MS media during early development (data not shown). After they were transferred to soil, transgenic plants appeared wild-type except for a slightly delayed flowering (~7-8 days late), when compared to WT plants (Figure 6A), indicating that over-expression of *JnRAP2-like* had no obvious deleterious effects on growth of the transgenic plants. We also observed that plants of the two OE lines showed a higher incidence of non-symmetric flowers (petals slightly zygomorphic) 40% (6-31) and 35% (14-16) vs. 0% (WT) (Figure 6A-2). Symmetric (actinomorphic) flowers are typical of the *Brassicaceae* [48]. 

### The Effects of Exogenous Plant Hormones on Plants Over-expressing 35S::*JnRAP2-like*

#### Ethylene

ERF family genes show a variety of ethylene and stress-regulated expression patterns [20,49-52]. To understand the effects of ethylene on the phenotype of *JnRAP2-like* OE lines, WT and transgenic plants growing on MS media were treated with 1-aminocyclopropane-1-carboxylic acid (ACC) (0, 25 and 100 μM) (Table 2). The constitutive ethylene response mutant *ctr1-1* was used as a positive control for ethylene-related phenotypes. On media without hormones, the length of hypocotyls of five-day-old, etiolated seedlings of OE transgenic lines 6-31 and 14-16 was 1.1 (SE=0.06) cm and 0.91 (SE=0.06) cm, respectively; slightly but significantly longer than WT hypocotyls [0.88 (SE=0.05)], P<0.05 (Figure 6B). The root length of the two OE lines was 1.0 (SE=0.06) and 0.79 (SE=0.08) cm, respectively, slightly but significantly shorter than the roots of WT plants [1.3 (SE=0.09) cm], P<0.05 (Figure 6B). After 25 and 100 μM ACC treatment, etiolated seedlings of the two OE lines showed inhibition of root and hypocotyl elongation that was not different from WT plants (Figure 6B). In short, both OE lines were similar to WT in response to ACC treatment. 

### Methyl Jasmonate

We treated OE lines with 50 µM MeJA to examine the effect of *JnRAP2-like* OE on JA-dependent signaling (Table 2). The jasmonate response mutant *jar1-1* mutant was used as a negative control for MeJA responses. After two weeks of growth on MS media supplemented with 50 µM MeJA, OE transgenic plants displayed highly branched, hairy roots with a less-developed primary axis, similar to the phenotype of the JA-insensitive *jar1-1*[53] http://www.arabidopsis.org/servlets/TairObject?type=germplasm&id=1005161577; Figure 6C); the vegetative development of the wild- type controls was severely stunted, with minimal root development, consistent with the observations of [51] (Figure 6C). The phenotypic differences among wild type plants, *jar1-1* mutant, and OE transgenic plants became more pronounced after three weeks (Data not shown). Higher concentration (200µM) of MeJA inhibited the growth of transgenic and wild-type plants one week after the treatment; after treatment with lower concentrations (10 and 25µM) of MeJA there was no obvious phenotypic difference between transgenic lines and WT plants (data not shown). 

The similarity we observed between the phenotype of *JnRAP2-like* OE plants and *jar1-1* plants may indicate that over-expression of *JnRAP2-like* produces a JA-insensitive phenotype, possibly because *JnRAP2-like* can function as a repressor of JA-dependent signaling in roots. *RAP2.6L* is maximally expressed in the maturation zone of roots [29], but its function in roots remains to be determined. 

In order to further characterize the effect of *JnRAP2-like* on ethylene-or JA-dependent defense responses, expression of two marker genes, *ChiB and PDF1.2*, was quantified in the leaves of transgenic lines. Real-time PCR showed lines 6-31 and 14-16 expressed *ChiB* at levels approximately 4.7- and 5.5-fold higher when treated with MeJA than without MeJA treatment, respectively, although wild type plants showed no significant change in expression of *ChiB* in response to 50 µM MeJA compared to untreated plants. Expression of *PDF1.2* in 6-31 and 14-16 transgenic lines in response to 50 µM MeJA was increased by 12- and 10-fold, respectively, when compared to transgenic plants without MeJA treatment (Figure 6D). This result was comparable to the 9-fold increase of *PDF1.2* expression in WT Col-0 treated with MeJA versus WT plants without MeJA treatment, indicating that *JnRAP2*-*like* in the OE lines were not expressing a neomorphic function. It has been shown that *RAP2.6L* is strongly induced by inoculation with *P. syringae* (Bio-Array Resource for Plant Functional Genomics; http://bar.utoronto.ca), so it is likely that *JnRAP2-like* functions to signal biotic stress. The small differences in induction between the two independent lines can probably be explained by differences in the location of the T-DNA insertion. It is also noted that the *PDF1.2* responses were in leaves, rather than roots. 

### Abscisic Acid

To determine if *JnRAP2-like* has a role in abscisic acid (ABA)-dependent signaling, plants of OE lines were germinated and grown on MS medium containing 0.1 µM ABA (Table 2). Results revealed that WT plants predictably failed to initiate photomorphogenic development [54], while OE transgenic lines produced fully expanded green cotyledons (Figure 6E). *afp1* mutant plants hypersensitive to ABA [55] were also used as controls for the response to ABA. Analysis of coexpression (http://www.cressexpress.org) showed that At3g56850 (AREB3), an ABA-responsive element binding protein, was the most highly co-expressed (r^2^ = 0.94) gene with *RAP2.6L* in roots of *Arabidopsis*; the second most highly co-expressed gene (r^2^ = 0.937), was the bZIP transcription factor-like *ABSCISIC ACID-INSENSITIVE5*. These results may indicate a role for *JnRAP2-like* in cross talk between ABA and other hormone responses.

### Ethylene and Methyl Jasmonate

A dose-response treatment of MeJA revealed that transgenic plants began to display mutant phenotypes two or three weeks after 50µM MeJA treatment. Higher concentration (200µM) of MeJA inhibited the growth of transgenic and wild-type plants one week after treatment. Treatment with lower concentrations (10 and 25µM) of MeJA resulted in no obvious phenotypic difference between transgenic lines and WT plants. To determine if ethylene and MeJA acted synergistically on the expression of *JnRAP2*-*like* as has been shown for other ERF genes [51], both ACC and MeJA were simultaneously added to MS media (Table 2). Results revealed that the treatment of transgenic lines with 10µM ACC and 25 µM MeJA phenocopied the root phenotypes associated with treatment with 50µM MeJA alone, indicating a synergistic interaction between ACC and MeJA(Data not shown), confirming the observation of Nakano et al. (2006b) [56] . 

## Discussion

Very few transcription factors associated with heartwood formation have been identified in hardwood trees. Here, we describe the isolation and characterization of a gene, *JnRAP2-like*, from black walnut, which belongs to B-4 group of the ERF subfamily in the AP2/ERF transcription factor super-family (Figure 3). Comparisons of sequence data indicated that *JnRAP2-like* was most similar to *RAP2.6L* of *Aradidopsis* (At5g13330). *JnRAP2-like* was highly abundant in the TZ of black walnut, especially in the fall; *RAP2.6L* is highly abundant in *Arabidopsis* xylem[29]. *JnRAP2*-*like* was also highly expressed in female flowers, as was observed for the poplar ortholog (PtpAffx.75787.2.A1_s_at) [31]. By over-expressing *JnRAP2-like* from black walnut in *Arabidopsis*, we discovered that *JnRAP2-like* is most likely a functional ortholog of *RAP2.6L*, and thus participates in the regulation of developmental events such as senescence/abscission and responses to biotic and abiotic stress. 

In our experiments, the phenotype of *35S::JnRAP2-like* transgenic roots and etiolated *Arabidopsis* hypocotyls was not different from WT plants when grown in the presence of abundant exogenous ACC (25 µM) (Figure 6B). This response was different from what was observed for other members of the ERF subfamily such as in tobacco [57], tomato [58], and *Thinopyrum intermedium* [59], where ERF family genes positively modulated a pronounced triple response. It has been pointed out that exposure to exogenous ethylene or increases in the synthesis of endogenous ethylene do not always induce ethylene responses in all tissue types or developmental stages [60]. Members of the *ERF* family regulate ethylene responses through the *cis*-acting sequence known as the GCC-box [26,27]. Many previous studies have shown that over-expressed ERF subfamily genes induce the transactivation of GCC-box containing genes to trigger a subset of responses related to biotic or abiotic stress [49,59-63]. It seems most likely from our results that *JnRAP2-like* does not directly participate in ethylene-triggered responses but rather in cross-talk between ethylene and MeJA, probably in a tissue-specific manner. *JnRAP2-like* OE transgenic plants treated with 50 µM MeJA showed highly branched, hairy roots with a poorly-developed primary axis (Table 2, Figure 6C). This phenotype in response to MeJA is mediated by ethylene [64]. But although the ethylene mediated response to MeJA in the OE transgenic plants was clearly discernable (loss of primary root axis, proliferation of root hairs), it was not as extreme as the phenotype of the WT plants (Figure 6C). This result was consistent with a *JnRAP2-like* mediated reduction in cross-talk between MeJA and ethylene in the roots. Conversely, in leaves, the increased expression of *ChiB* in *JnRAP2-like* OE transgenic plants treated with 50 µM MeJA indicated that *JnRAP2-like* may synergistically activate defense-related gene expression either directly or indirectly in response to injury or ethylene. Lorenzo et al. (2003) [51] showed that *ChiB* was only weakly responsive to MeJA treatment alone, but that MeJA in combination with ethylene produced about a 4x spike in expression levels. We showed that leaves of OE transgenic plants expressing *JnRAP2-like* responded to MeJA treatment by producing about 4x more *ChiB* transcripts than leaves from WT plants. We concluded that in leaves, unlike roots, *JnRAP2-like* may act in concert with MeJA signals to amplify stress responses. It has been shown that *RAP2.6L* is involved in the regulation of ethylene-dependent abscission [30,33]. Strong evidence for a relationship between RAP2.6L and ethylene is presented in [65]. The relationship between RAP2.6L and JA is further confirmed by [66]. We observed a slight up-regulation of *JnRAP2-like* in yellow, senescent leaves, as compared to green leaves, but it is not clear if this was in response to short days [32], an ethylene/senescence-related transcriptional program [67], or both. 

In *Arabidopsis*, *PDF1.2* encodes a plant defensin commonly used as a marker for characterization of the ethylene- or JA-dependent defense responses [26,68]. We observed strong induction of *PDF1.2* in OE transgenic plants in response to MeJA. The response of the OE plants was about the same as WT leaves (about 10x increase in *PDF1.2* transcript). So *PDF1.2* was unlike *ChiB* in that we saw no evidence of a synergistic interaction where *JnRAP2-like* expression enhanced responses to MeJA, much as ethylene treatment would. It is possible, however, that we saturated the *PDF1.2* expression response, i.e., that we did not see a synergistic response to MeJA in the OE transgenic plants because the plants were already making as much *PDF1.2* as possible (> 30 fold over baseline, Figure 6D).

Lorenzo et al. (2003) [51] suggested a model whereby *ERF1* (and possibly other ERFs) receives signals from both the ethylene and JA pathways leading to pathogen resistance, and there is evidence that *RAP2.6L* is strongly induced in both compatible and incompatible interactions with downy mildew (*Hyaloperonospora parasitica*) [69]. Combining the results described above with the observed synergism resulting from co-treatment of *JnRAP2-like* OE plants with 10µM ACC and 25 µM MeJA [also reported for *RAP2.6L* by [56], we propose that *JnRAP2-like* participates in the integration/crosstalk of ethylene and jasmonate signaling pathways, as was observed by [70] for the *ERF ORA59* (= *AtERF15*), and numerous other ERFs [71], although the role of *JnRAP2-like* in crosstalk between ethylene and MeJA signals may be different in roots versus aerial tissues, or even tissue-specific.

We found that germinating seedlings of *JnRAP2-like* transgenic plants continued growing when treated with 0.1 µM ABA, while the WT plants did not, indicating that *JnRAP2-like* may also negatively regulate an ABA-dependent signaling pathway in roots (Figure 6E). Previous research showed that *AtERF3*, *-4*, and *-7* negatively regulated ABA-responsive genes as transcriptional repressors through chromatin modification or protein degradation [70-72]. These results may indicate that *JnRAP2-like* can play a role in responses to ABA. It is not known if ABA is present in a concentration gradient in xylem, or if there is a relationship between ABA and heartwood formation, although ABA has been studied in relation to plant abiotic stress responses, such as cold and drought. It has also been suggested that *RAP2.6L* was highly expressed in xylem tissue, and its expression was related to stress (Genevestigator V3; 250287_at). ABA also mediates (seed) maturation and dormancy [73]. Further analysis of the relationship between ABA signaling and *JnRAP2-like* is needed, since it is also possible the ABA-related phenotypes we observed represent a neomorphic function of *JnRAP2-like* in the OE plants [74].

### The possible function of *JnRAP2-like* during heartwood formation in black walnut

Heartwood formation is a complex developmental process whereby environmental cues (e.g., temperature, day-length) are converted to internal signals (i.e., hormones) that are communicated to a small number of xylem parenchyma cells that execute a PCD cascade [5]. The result is similar to the response of conifer cells to wounding [22], in which a MeJA signal is amplified by ethylene, leading to activation of enzymes that are related to phenolic biosynthesis, the accumulation of phenolic extractives, and heartwood formation [8,15,75,76]. This observation may indicate that heartwood formation is related to xylem defense pathways that serve to compartmentalize injury from biotic agents that damage xylem [77]. ERF subfamily genes are typically activated in response to biotic and abiotic stress and, in turn, they activate responses through ethylene-, JA-, and ABA-dependent pathways [26,52,70]. For instance, *JERF* mediates ethylene, MeJA, and ABA signals [78]. These same hormonal signals have been shown to influence defense responses in trees and the reprogramming of parenchyma cells to accumulate polyphenolic compounds [16,19,22] such as those accumulated in black walnut heartwood [15]. *Arabidopsis* RAP2.6L strongly and consistently co-expresses with cinnamyl alcohol dehydrogenase [79] (see http://atted.jp), possibly providing a connection between JNRAP2-LIKE and the accumulation of phenolic compounds such as those found in heartwood. Linkage between JnRAP2.6L and heartwood formation, if present, awaits more definitive experimental results [66]. 

Taken together, these previous observations point to the involvement of ERFs that modulate cross-talk between ethylene and JA signaling in the development of heartwood. We showed that *JnRAP2*-*like* is expressed in the TZ of black walnut and is upregulated in autumn, when it is believed that heartwood is formed [4,8-10]. Whether *JnRAP2-like* is actually involved in the signal transduction for heartwood formation cannot be demonstrated until a more complete understanding of how heartwood forms is available. 

## Supporting Information

Figure S1 **Southern blot result for the copy number of *JnRAP2-like* in black walnut.** Two Enzymes, Xhol and EcoRV, were used for digestion of the genomic DNA in black walnut. 2.7kb was used for positive control.
(PPT)
