# Supplementary material for: Roles of JnRAP2.6-like from the Transition Zone of Black Walnut in Hormone Signaling
Source: PLoS One. 2013 Nov 12;8(11):e75857. doi: 10.1371/journal.pone.0075857 (PMC3827044; doi:10.1371/journal.pone.0075857)
Supplement: Figure S1 — Southern blot result for the copy number of JnRAP2-like in black walnut. Two Enzymes, Xhol and EcoRV, were used for digestion of the genomic DNA in black walnut. 2.7kb was used for positive control. (PPT) [file pone.0075857.s001.ppt]

## Slide 1
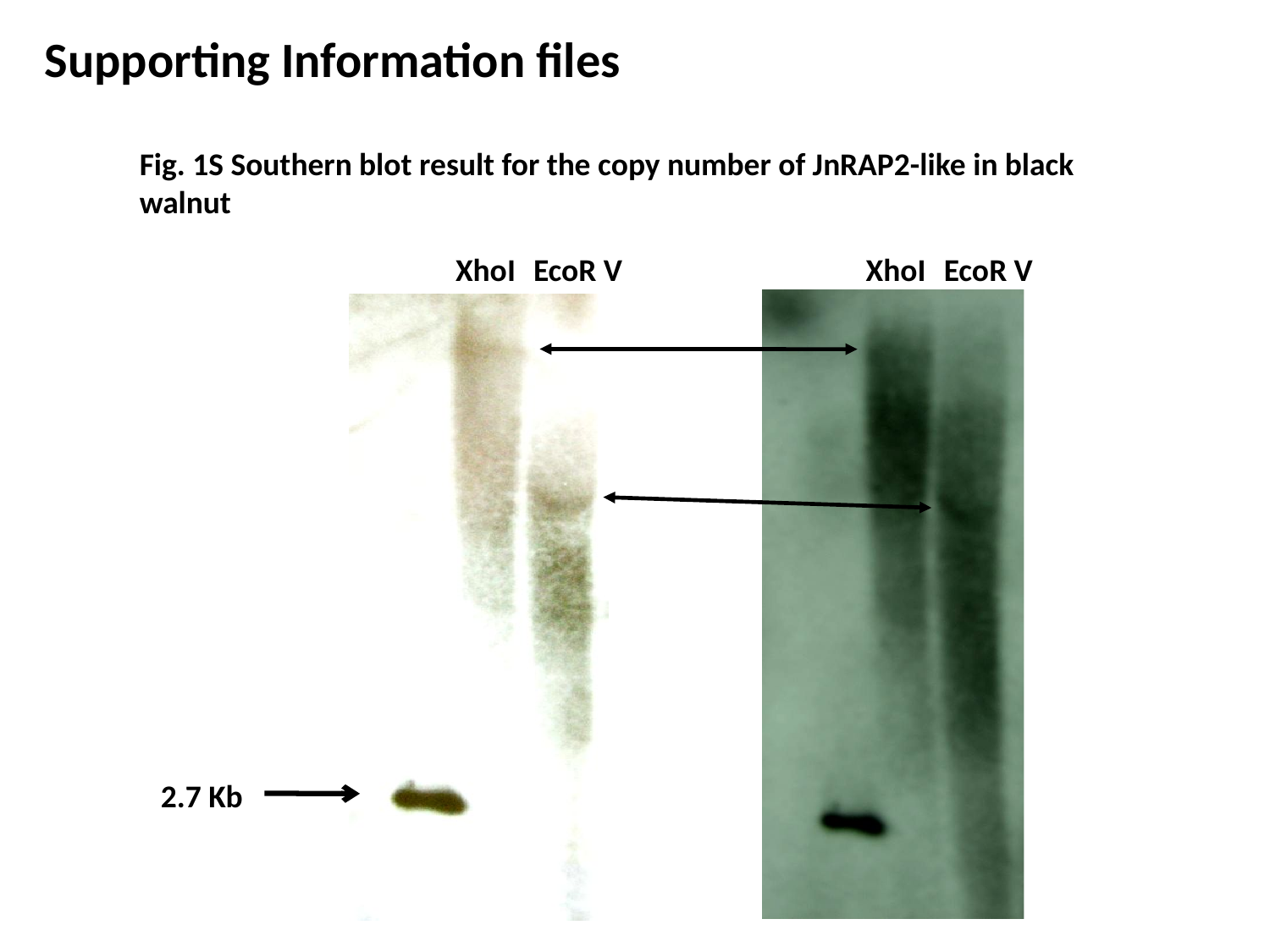

Supporting Information files
Fig. 1S Southern blot result for the copy number of JnRAP2-like in black walnut
XhoI
EcoR V
XhoI
EcoR V
2.7 Kb
